# Preference of caregivers on residential care homes for older persons with versus without communication problems: a discrete choice experiment

**DOI:** 10.1186/s12877-022-03073-9

**Published:** 2022-05-10

**Authors:** Kailu Wang, Eliza Lai-Yi Wong, Angel Hor-Yan Lai, Carrie Ho-Kwan Yam, Ethan Ming-Yin Ip, Annie Wai-Ling Cheung, Eng-Kiong Yeoh

**Affiliations:** 1 Centre for Health Systems and Policy Research, The Jockey Club School of Public Health and Primary Care, Faculty of Medicine, The Chinese University of Hong Kong, Hong Kong SAR, China; 2 Department of Applied Social Science, Hong Kong Polytechnic University, Hong Kong SAR, China

**Keywords:** Elderly care, Long-term care facility, Consumer-directed care, Conjoint analysis, Preference

## Abstract

**Background:**

A residential care service voucher scheme has been introduced to expand the capacity and enhance choice of public-funded long-term care (LTC) in Hong Kong, enabling users to have greater choices over the types of LTC facilities. Older persons with communication problems have difficulties in understanding the care options available to them and expressing their preferences to care homes and daily service delivery, therefore hindering their ability to exercise control and choice. Thus, they may have different needs and preferences for the facilities than others due to their conditions. This study therefore aimed to investigate their preference for care homes in comparison with those without communication problems.

**Methods:**

A discrete choice experiment was conducted to elicit preference for six attributes derived from prior studies. The family caregivers of a random sample of older voucher holders were invited to undertake face-to-face interview. Willingness-to-pay (WTP) for the attributes was estimated for those with or without communication problems separately.

**Results:**

Two hundred eighty-three caregivers provided valid responses (74% response rate). Caregivers of those with communication problem preferred facilities operated by non-governmental organizations (WTP=HK$1777.4) and shorter travelling time (WTP=HK$1502.5 for <=0.5 hours), while those without the problem had greater preference for fewer roommates (WTP=HK$3048.1 for single room) and extra healthcare professionals (WTP=HK$1765.5). Heterogenous WTPs were identified from those with different income, marital status and caregivers’ age.

**Conclusions:**

The reputation, greater space and staff, and proximity/familiarity of the facilities were important for those with communication problems. To help meet these preferences, the facilities could establish collaborations with local community-based service providers and build their own outreach team to familiarize themselves with older persons. Additionally, household income and informal support availability should be considered for care planning.

**Supplementary Information:**

The online version contains supplementary material available at 10.1186/s12877-022-03073-9.

## Introduction

The decreasing fertility rate and increasing life expectancy are leading to an ageing population worldwide, which significantly increases the needs for long-term care (LTC) [1, 2]. In line with this trend, the sustainable provision of public-funded LTC service in Hong Kong, China is threatened by increasing demands for LTC, including the residential care services provided by Care and Attention Homes for elderly with poor health and physical impairment and mild mental disabilities, and Nursing Home for those with severer physical and mental impairment. The waiting time for the government subsidized residential care homes is long, with an average of waiting time of 42 months as of March 2021 reflecting large unmet demand among the older persons in need of this service [3, 4]. In Hong Kong, the publicly-funded LTC provides inexpensive services with relatively higher average quality of care, while the quality in private sectors varied across services with a wide range of prices. Most older persons cannot afford expensive private services with good quality of care, so they have to apply and usually endure long waiting time for public services.

To meet the demand for LTC, public-private partnership (PPP) is one of common tools employed by the government of Hong Kong to expand the capacity of LTCs, while ensuring an affordable price. A means-tested residential care service voucher (RCSV) pilot scheme was introduced into local LTC to improve the private service provision and utilization, which provides benefits to service users in the form of a voucher for them to choose and purchase the residential care services that meets their needs and preference [5, 6]. This scheme adopted a “money-following-users” approach, which is similar to the design of LTC services provided under self-directed/consumer-directed care or “personal budgets” approach in other countries and regions, and enables the users to gain greater control and choice in deciding the services for themselves [5, 7–9]. Apart from the fact that most schemes of this kind are implemented in home- and community-based care settings, the Direct Payments (DPs) in the UK has also been piloted in residential care as a mechanism to promote control and choice for service users [10]. A qualitative evaluation of this programme revealed that some of the users welcomed the opportunities to access different services and empowerment for controlling the budget, while others were expecting more relevant choices for the services [11]. The evaluation findings highlighted the needs of service users for greater choices in residential care.

For RCSV, the eligibility of older persons needs to be ascertained by standardized needs assessment based on their physical and mental function for public-funded LTC, and eligible users need to make different levels of co-payments for the voucher that are determined based on means test [6]. On the supply side, there are different types of providers of LTC services with the eligibility of service provision for RCSV scheme, including private for-profit care homes that meet certain standards in pre-capita space and staff number, and the government subsidized care homes and self-financing homes with residential places not subsidized by public funds and are run by non-governmental organizations (NGOs) [6, 12]. These NGO homes have greater average net floor area and number of staff than private for-profit care homes and they are usually deemed by general public to have higher overall quality of care as well [13].

Since the users under the RCSV scheme are empowered to make their own service choice, we need to understand their preferences. It is important for older persons to express their own preferences for care homes and be able to understand what other people say, such as case managers and social workers, so that they can be provided with appropriate and tailored options of private care home [14, 15]. This requires older persons to have sufficient communication abilities, including absence of hearing difficulties, abilities to understand others, and the ability to make oneself understood by the others. However, the prevalence and complexity of communication problem increases with age, due to the increasing frailty of the senses [16]. This not only hinders their ability to exercise control and choice in selecting the most suitable care homes, but also affects their ability to make daily-life decisions when living in the care homes. Thus, preferences for the care homes could be different between those with communication problems and those without such problems. Under this circumstance, family caregivers usually play a crucial role in helping older persons with communication problem to make suitable care decisions [17], especially when it is difficult for them to express their needs and understand the options under the RCSV scheme [18, 19]. The preferences of these family caregivers become useful information for case managers and social workers to understand their clients’ needs and also for private service providers to adjust their services and information dissemination strategies, in order to achieve a greater efficiency in matching the residential care services to the users, while disparities in preference and decisions between family caregivers and the older persons should be taken into account in obtaining relevant preference [20]. Furthermore, the application of means-tested and co-payment mechanisms in voucher services has been implemented as one of first trials in the world. While innovative, we need more information to understand users’ willingness to pay in order to adjust the co-payment levels of the vouchers to increase its uptake.

Considering the importance of users’ preferences for care homes, this study aimed to elicit the stated preference on care homes (i.e. LTC facilities) of family caregivers for the older service users with versus without communication problems of RCSV scheme who usually serve as surrogates in LTC decision-making for older persons. We hypothesized that 1) the preference of caregivers of older persons with communication problems may be different from those without such problems (hypothesis 1); and 2) the preferences between these two groups of caregivers may be associated with the financial status and availability of informal caregiver support as reflected by household income, and the marital status of the older person and family caregiver’s age, respectively (hypothesis 2). The willingness-to-pay for care homes were also estimated based on their responses.

## Methods

A discrete choice experiment (DCE), also known as choice-based conjoint analysis, was conducted among older adults living in residential care homes and their informal caregivers during January 2019 to June 2019 to elicit their preference on the care homes. DCE is a questionnaire-based quantitative method used to uncover stated preference (SP) on goods described by a series of attributes [21, 22]. Compared with methods to elicit revealed preference (RP), which is estimated based on data collected from actual market behaviours, DCE allows examinations of preference for future products or services that are not yet available in the market using choice alternatives described by hypothetical combinations of attribute levels [21]. Compared with other methods to estimate SP, such as contingent valuation, DCE can simulate choice behaviours that consumers perform in real-world decision-making [23]. DCE is gaining popularity in research of LTC services, including research on quality of life [24, 25], LTC insurance [26, 27], and care coverage [28].

### Sampling and data collection

The target population of the DCE was the voucher holders, including those who were holders at the time of study and prior holders who withdrew from the scheme after using the voucher for a while, under the RCSV pilot scheme. This was because they had experience of making choices over different care homes, which is important for the respondents to be aware of their own preference and provide relevant and rational responses to the DCE tasks. Records of all the voucher holders (*N* = 622) on their basic demographic data and other information about their participation in the scheme were obtained on the 31 October 2018 from the Social Welfare Department (SWD) who oversees the vouchers services. An anonymized identifier was assigned to each individual in this dataset, and a simple random selection procedure was performed in this dataset to select around 400 individuals for further contact. Identifiers of these selected individuals were returned to SWD to obtain their contact information. These individuals were then approached on telephone for initial consent to participate in an interview with references from their case managers.

The interview was conducted on a face-to-face basis by trained interviewers with a questionnaire in Chinese at residential care facilities or their home. As most of the older adults who joined the scheme suffered from diminishing cognitive capacity or mental illness, the interview was answered by their family caregivers who were the decision-maker for the LTC relevant decisions of the older individuals. Ethics approval was obtained from the Survey and Behavioural Research Ethics Committee of The Chinese University of Hong Kong prior to commencement of this study. Written informed consent was obtained before the interview and each one lasted around 30 minutes.

### Attributes and levels

In a DCE, the respondents are presented with several choice tasks with two or more alternatives. Each alternative is described by combination of a series of attributes and their levels. These attributes and levels were determined according to a prior survey on factors affecting choices over service providers of RCSV among its potential users [5], as well as a focus group discussion among LTC service users and social care professionals. Findings revealed that the older adults would consider the following attributes when deciding on which residential care homes they prefer: quality of care in the care home; featured with availability and quality of care staff and medical services; proximity of the facility to their family; and environment of the facility. Besides this survey, a focus group discussion among the older adults with experience of using RCSV and managers and staff of care homes indicated that the older adults would consider number of people sharing the room as an important factor in choosing the care homes, and older adults would praise the choice and flexibility of services in the care homes.

The attributes for this DCE were then designed based on the aforementioned responses, namely type of the care home, distance from the care home to their family, number of people sharing the room, availability of medical personnel, flexibility of services, and monthly co-payment amount (Table 1). Type of the care home denoted who operate the facilities, including subvented/contract/ self-financing homes run by NGOs (referred to as NGO care homes below) and private for-profit homes (referred to as private home below). The monthly co-payment amount for the care home, a monetary variable indicating the actual out-of-pocket payment for each care home alternative, was incorporated for estimating willingness to pay (WTP) and the values were set based on actual co-payment amount in RCSV scheme.

**Table 1:** Attributes and levels of care homes for the discrete choice experiment

Attributes	Level 1	Level 2	Level 3	Level 4
Type of care homes	Private-operated (for-profit) care homes	NGO care homes: subvented/contract/ self-financing care homes	–	–
Distance (travelling hour to the facility from home/ office)	More than an hour	Half an hour to one hour	Within half an hour	–
Room type	Shared room (4 or more people)	Shared room (2–3 people)	Single room	–
Manpower	Extra care workers compared with standard requirement	Extra healthcare professionals compared with standard requirement	–	–
Enhanced services	Limited choices in enhanced services	Flexibility of choices for enhanced services	–	–
User monthly co-payment amount	HK$665	HK$1329	HK$2657	HK$3986

### Experimental design

There was a total of 288 combinations (2 × 3 × 3 × 2 × 2 × 4) of all attribute levels, and therefore 41,328 pairwise choice sets, which is not practically feasible to put in the questionnaire. For the DCE, D-optimal algorithm was used to selected 60 choice sets with 2 alternatives in each of them from a full-factorial design of all attribute levels [21], with priors of coefficient of each attribute level set to improve the efficiency of the design. The D-efficiency score achieved 0.491 for this design. Only two alternatives were included for each choice set to reduce the cognitive burden of respondents. A pilot test among 5 older adults was conducted to find out if the wording and design of the questionnaire was appropriate, and remove unrealistic choice tasks. Based on the pilot test, the suitable number of choice tasks for each should be 4 tasks so that they would not cause severe cognitive burden, as some of the family caregivers were older adults and potentially had diminishing cognitive capacity to answer the questionnaire. An opt-out option was added to each of the choice sets in addition to the two alternatives, which was framed as opting-out from the two care home alternatives and waiting for 1–3 months for the staff to show you other care homes. To reduce cognitive burden while covering all selected choice tasks, the 60 choice sets were randomly assigned to 15 blocks. Each respondent only needed to answer 4 choice tasks in one of the blocks that they were randomly assigned to. An exercise choice set was added for each respondent prior to formal tasks for them to understand the DCE choice tasks and its attribute levels.

### Measurements

Apart from the DCE, the socio-demographic and health-related information of the older persons and their family caregivers in a Minimum Data Set – Home Care (MDS-HC) 2.0 questionnaire were extracted from administrative system of SWD. Socio-demographic information included age, sex, marital status, and age, household income, and education level of the caregivers. Those with household income below HK$15,000, around half of median monthly household income level in Hong Kong, which is a poverty line set by the government, were considered to have “lower income” [29, 30]. Health-related information included 8 items of Activities of Daily Living (ADLs) performance and cognitive impairment (including at least one of short-term memory loss, decision-making ability impairment, change in mental function, and agitation or disorientation in past 90 days) of the older persons, and data item used to assess their communication abilities. According to its manual, communication problem is characterized as having either hearing difficulties, problems in making oneself understood, or problems in understanding others. A binary variable was used to categorize those with or without communication problems.

### Statistical analysis

The random utility theory forms the basis of analysis DCE responses. The utility (U) of individual i choosing a care home comprises of a deterministic component (V) and a stochastic component (ε). The deterministic component V is a function of the DCE attribute levels (A_k_):

$${U}_{k,i}={V}_{k,i\mid opt- in}+{\upvarepsilon}_i=\kern0.5em \sum_{k=0}^K{\upbeta}_k\ {A}_{k,i}+{\upvarepsilon}_i$$

$$=\beta 0+\beta 1\bullet TYPEngo +\beta 2\bullet DISTANCE0.5\sim 1 hour+$$

$$\beta 3\bullet DISTANCE<0.5 hour+\beta 4\bullet ROOM2\sim 3 ppl+$$

$$\beta 5\bullet ROOMsingle+\beta 6\bullet MANPOWERhealthcare+$$

$$\beta 7\bullet SERVICEchioce+\beta 8\bullet PRICE+\varepsilon$$

$${V}_{i\mid opt- out}=0$$

The attribute levels that are dummy-coded were independent variables. As the utility cannot be measured directly, their choices to the care home alternatives (binary variable: yes or no) in DCE constituted the dependent variable. The responses from DCE were analyzed by multinomial logistic (MNL) regression, and the opt-out option was included in the model as an alternative-specific constant (ASC, β_0_) [31]. Stata 15.0 was used for data analysis. To address hypothesis 1, the preferences for the attribute levels were tested along with their interaction term with the communication problem. To better interpret the regression outcomes, postestimation of the attribute coefficients for those with or without communication problems were performed by adding the coefficients of the attribute main effects and the coefficients of the interaction terms, and the 95% confidence intervals were estimated based on Delta method (original model outcomes reported in supplementary Tables S6, S7, S8 and S9). A latent class logit (LCL) model was then applied to account for the observed and unobserved preference heterogeneity and their association with communication problems, and ADL and cognitive impairment for identifying the difference in preference across respondents with different physical and psychosocial health status. Number of classes was determined based on the Bayesian information criterion (BIC) of the model [32]. To address hypothesis 2, the preferences were also examined among these subgroups using interactions between the DCE attributes and household income, marital status of the older persons and age of their caregivers, which constitute indicators for availability of informal care in MNL model. Another LCL model was also used to examine the preference heterogeneity across these factors along with communication problem. Those with missing values in communication problems were excluded from analysis. Using the MNL regression coefficient estimation, the probability of choosing the care home with certain attribute levels ***A***_***k***_ were estimated by exp.(V_k_)/(1 + exp.(V_k_)). Their marginal willingness-to-pay (MWTP) were calculated based on regression outcome by dividing the coefficient of a specific attribute level k (1 ≤ k ≤ 7) by the coefficient of their co-payment amount (β_8_):

$$MWTP={\beta}_k\left/{\beta}_8\right.$$

As sensitivity analysis, a third LCL model without any membership variables (i.e., communication problem, household income, marital status, and caregivers’ age) were applied to show the class segmentation based on the preference for the attributes only, and the posterior probability of class membership were then estimated for each individual. The relationship between the probability to be in any of the classes and these membership variables were examined in multiple linear regressions.

## Results

### Characteristics of respondents

Of the 407 older voucher holders who were invited to participate, 301 completed the DCE questionnaire with a response rate of 74%. The majority of the people who did not participate were not reached by telephone (*n* = 97), while 8 of them rejected the interview and 1 older person passed away. Among 301 participants, 18 of them were older person and 283 were family caregivers. As older persons were too few to be analyzed, only responses from 283 family caregivers were analyzed to ensure the homogeneity of the study sample (Table 2). Among these caregivers, the majority of the older persons that they were taking care of were aged 80–99 years (85.8%), 71.0% were female, and 23.7% were married. As for health status of the older persons, 97.2% reported to have impairment in ADLs, 91.9% had cognitive impairment, and 80.6% had communication problems. For the caregivers themselves, 51.6% of them were aged 60 years or above. Around 62.6% received education higher than high school, and 60.8% had income levels lower than HK$15,000, around half of median monthly household income level in Hong Kong, which is a poverty line set by the government [29, 30]. Most of the aforementioned characteristics were not significantly different between those with and without communication problems, except cognitive impairment (*P* = 0.002).

**Table 2:** Socio-demographic and health-related characteristics of the participants

	Without communication problem	With communication problem	Total	*P* value
**Age group**				
60–69	0 (0.0)	8 (3.5)	8 (2.8)	0.162
70–79	9 (16.4)	18 (7.9)	27 (9.5)	
80–89	27 (49.1)	101 (44.3)	128 (45.2)	
90–99	18 (32.7)	97 (42.5)	115 (40.6)	
100+	1 (1.8)	4 (1.8)	5 (1.8)	
**Sex**				
Male	20 (36.4)	62 (27.2)	82 (29.0)	0.178
Female	35 (63.6)	166 (72.8)	201 (71.0)	
**Marital status**				
Unmarried	40 (72.7)	176 (77.2)	216 (76.3)	0.484
Married	15 (27.3)	52 (22.8)	67 (23.7)	
**Activities of daily living**				
No impairment	1 (1.8)	7 (3.1)	8 (2.8)	0.615
With impairment	54 (98.2)	221 (96.9)	275 (97.2)	
**Cognition**				
No impairment	10 (18.2)	13 (5.7)	23 (8.1)	0.002
With impairment	45 (81.8)	215 (94.3)	260 (91.9)	
**Carer’s age**				
Below 60 years	28 (50.9)	109 (47.8)	137 (48.4)	0.679
60+ years	27 (49.1)	119 (52.2)	146 (51.6)	
**Carer’s education level**				
Primary school or below	12 (21.8)	33 (14.5)	45 (15.9)	0.299
Secondary school	10 (18.2)	50 (21.9)	60 (21.2)	
High school	17 (30.9)	95 (41.7)	112 (39.6)	
Higher education	15 (27.3)	50 (21.9)	65 (23.0)	
(missing)	1 (1.8)	0 (0.0)	1 (0.4)	
**Carer monthly household income**				
< HK$ 15,000	30 (54.6)	142 (62.3)	172 (60.8)	0.220
HK$ 15,000+	19 (34.6)	60 (26.3)	79 (27.9)	
(missing)	6 (10.9)	26 (11.4)	32 (11.3)	
**Total**	55 (100.0)	228 (100.0)	283 (100.0)	

The median interview length was 25 minutes (interquartile range [IQR]: 18–60 minutes). There were a small and comparable proportion of respondents who consistently chose all the first alternatives (i.e., on the left-hand side) (*n* = 19, 6.7%) and all the second alternatives (i.e. on the right-hand side) (*n* = 21, 7.4%) in all four choice sets. The percentage of those choosing the first alternative was 50.4%, which is similar to the percentage of choosing the second alternatives (47.5%). There were 24 out of 1132 choice tasks (2.1%) selecting the opt-out option. Only 2 respondents consistently chose opt-out. The likelihood of opting-out was stable and slightly reduced from the first choice set (*n* = 7, 2.5%) to the fourth choice set (*n* = 4, 1.4%).

### Preference on the care homes according to communication abilities

The MNL results of the DCE for older persons with or without communication problems are shown in Table 3. Both groups of respondents attached significantly greater importance to the room type and had similar WTP for greater flexibility of enhanced services. The caregivers of older persons without communication problems were found to have higher WTP for extra healthcare professionals than those with the problem (HK$1386.8 vs no significant preference, *P* = 0.010) (US$1 = HK$7.8); while the latter groups of respondents had significant preference (i.e. the WTP is significantly higher than zero) for NGO care homes (HK$1777.4) and for within half-hour travel (HK$1502.5) from the care facility to their home, but they were not significantly higher than the preference of those without the problem.

**Table 3:** Preference and willingness-to-pay for the attributes of care homes according to communication abilities of older persons

Attributes	Older person without communication problems			Older person with communication problems			*P* value^a^
	Coeff.	95%CI	MWTP	Coeff.	95%CI	MWTP	
Type of care homes: NGO care homes	0.24	(− 0.15, 0.64)		0.47*	(0.26, 0.68)	1777.4	0.316
Distance: half an hour to one hour travelling time	0.17	(− 0.25, 0.60)		0.27*	(0.07, 0.48)	1034.9	0.672
Distance: Within half an hour travelling time	0.15	(−0.32, 0.62)		0.40*	(0.15, 0.65)	1502.5	0.358
Room type: shared room (2–3 people)	0.63*	(0.20, 1.07)	2082.4	0.33*	(0.13, 0.53)	1240.8	0.213
Room type: single room	0.93*	(0.34, 1.51)	3048.1	0.54*	(0.27, 0.81)	2043.2	0.241
Manpower: extra healthcare professionals	0.54*	(0.22, 0.85)	1765.5	0.09	(−0.05, 0.23)		0.010*
Enhanced service: flexible choice of enhanced services	0.42*	(0.10, 0.74)	1386.8	0.33*	(0.13, 0.53)	1554.2	0.954
Copayment (per HK$1000)	−0.30*	(− 0.47, − 0.14)		− 0.26*	(− 0.35, − 0.18)		0.676
Opt-out	−2.27*	(−3.30, −1.25)		−2.81*	(−3.36, −2.26)		0.366

^*^P < 0.05; the reference levels of the attributes are: 1) Type of care homes: private for-profit homes; 2) Distance: Over one hour travelling time; 3) Room type: Shared room (4–6 people); 4) Manpower: more care workers; and 5) Enhanced service: limited choices in enhanced services

^a^The *P* values in this column that were derived from the interaction terms of the regression model indicate the difference of coefficient estimates of the same attribute between those with and without communication problems. The coefficients in the table were calculated by adding the coefficients of the attribute main effects and the coefficients of the interaction terms between the attribute and communication problem. The 95% confidence intervals were estimated using Delta method. Original model outcomes can be found in supplementary Table S6

In the worst-case scenario, the probability to choose a private-operated care home with over one-hour travelling time, 4+ people shared room, extra care workers rather than healthcare professionals, limited choices in enhanced services and the HK$3986 monthly co-payment amount was 22.9% (without communication problem) / 25.8% (with communication problem). The probability to choose an NGO home with less than half-hour travelling time, single room, extra health professionals, various choices for enhanced services, and no copayment was 90.6% for those without communication problem and 87.1% for those with this problem. If the same care home is private-operated instead of NGO, the probability was 88.4% for those without communication problem and 80.8% for those with communication problem. If the same care home only has 4+ people shared room, the probability to choose such a care home was 79.4% (NGO-operated)/ 75.1% (private-operated) for those without communication problem and 79.7% (NGO-operated)/ 71.0% (private-operated) for those with communication problem.

The result from the LCL model (Table 4) reveals that substantial preference heterogeneity across individuals. Class 1 (class share 64.3%) represents those with preference for NGO care homes, shorter distance, and rooms with fewer roommates who were also sensitive to the copayment amount. Class 2 (3.0%) represents those with specific preference for single room and higher likelihood to opt-out. Class 3 (32.7%) represents those who preferred private-operated home. It was also found that caregivers of older persons with communication problems were more likely to be in class 1, which is consistent with the findings in MNL model shown in Table 3, while there was no significant difference between those with and without ADL impairment or cognitive impairment.

**Table 4:** Preference heterogeneity across physical and psychosocial health status in latent class model

	Class 1		Class 2		Class 3	
	Coeff.	95%CI	Coeff.	95%CI	Coeff.	95%CI
**Attribute levels**						
Type of care homes: NGO care homes	0.78*	(0.56, 0.99)	0.97	(− 0.16, 2.10)	−1.13*	(− 2.10, − 0.17)
Distance: half an hour to one hour travelling time	0.38*	(0.18, 0.57)	0.58	(−0.69, 1.85)	− 0.16	(− 0.93, 0.61)
Distance: Within half an hour travelling time	0.58*	(0.33, 0.83)	0.80	(−0.57, 2.17)	0.00	(−1.19, 1.19)
Room type: shared room (2–3 people)	0.46*	(0.26, 0.67)	0.94	(−0.60, 2.48)	−0.34	(−1.13, 0.44)
Room type: single room	0.63*	(0.35, 0.90)	2.15*	(0.53, 3.77)	0.75	(−0.66, 2.16)
Manpower: more healthcare professionals	−0.03	(−0.17, 0.12)	0.41	(−0.63, 1.45)	6.21	(−113.34, 125.76)
Enhanced service: flexible choice of enhanced services	0.01	(−0.16, 0.19)	0.77	(−0.30, 1.84)	7.12	(− 112.48, 126.72)
Copayment (per HK$1000)	−0.37*	(− 0.45, − 0.29)	0.08	(− 0.35, 0.52)	0.35	(− 0.07, 0.78)
Opt-out	−3.92*	(−4.73, − 3.11)	4.54*	(2.36, 6.71)	−5.62	(− 745.72, 734.48)
Class share	0.643		0.030		0.327	
**Membership**						
Communication problem	0.79*	(0.12, 1.46)	−0.04	(−1.22, 1.14)	Ref	–
ADL impairment	−1.01	(−5.06, 3.03)	−2.93	(−6.82, 0.95)	Ref	–
Cognitive impairment	0.26	(−0.68, 1.20)	−0.54	(−1.93, 0.84)	Ref	–
Obs	3396					
Log likelihood	− 757.976					
BIC	1515.952					

*P < 0.05; the reference levels of the attributes are: 1) Type of care homes: private for-profit homes; 2) Distance: Over one hour travelling time; 3) Room type: Shared room (4–6 people); 4) Manpower: more care workers; and 5) Enhanced service: limited choices in enhanced services

In the sensitivity analysis (supplementary Table S4), the pattern of preference heterogeneity is similar to the model reported in Table 4. Class 1 (62.4%) represents those with preference for NGO care homes, shorter distance, and rooms with fewer roommates and those who were sensitive to copayment amount. Class 2 (2.6%) represents those with preference for single room and high up-out likelihood. Class 3 (35.0%) represents those with preference for private-operated home, more healthcare professionals and flexible enhanced services. Based on the multiple regressions for the class membership probabilities (supplementary Table S5), those with communication problems were more likely to be in class 1 (adjusted difference in probability: 0.07, 95%CI: 0.04–0.10), and less likely to be in class 2 (adjusted difference in probability: -0.02, 95%CI: − 0.03 - -0.01) and class 3 (adjusted difference in probability: -0.05, 95%CI: − 0.08 - -0.02) than those without communication problem.

### Preference on the care homes according to income, marital status and caregiver’s age

The DCE results of preference according to household income level, marital status and age of the caregivers are shown in Table 5. First, among the lower income group (*n* = 172), the caregivers preferred NGO care homes (HK$1407.4), shorter distance (< 0.5-hour travel: HK$1681.7), fewer roommates (single room: HK$1357.0), and flexible enhanced services (HK$850.0). In the high income group (*n* = 79), the caregivers preferred NGO care home (HK$1418.3), single room (HK$1726.6) and flexible enhanced services (HK$2345.2). Comparing these two groups, caregivers in the high income group were found to have significantly greater preference for flexible enhanced services than those in the low income group (HK$2345.2 vs HK$850.0, *P* = 0.037). No significant differences were found in preferences for the other attributes.

**Table 5:** Willingness-to-pay (HK$) for the attributes of care homes according to income, marital status, and caregiver’s age

	Lower income (<HK$15,000) (*n* = 172)			Higher income (HK$15,000+) (*n* = 79)			*P* value^a^
	Coeff.	95%CI	MWTP	Coeff.	95%CI	MWTP	
Type of care homes: NGO care homes	0.41*	(0.14, 0.69)	1407.4	0.33*	(0.06, 0.61)	1418.3	0.676
Distance: half an hour to one hour travelling time	0.20	(− 0.08, 0.48)		0.13	(−0.14, 0.40)		0.724
Distance: Within half an hour travelling time	0.50*	(0.17, 0.82)	1681.7	0.10	(−0.23, 0.44)		0.101
Room type: shared room (2–3 people)	0.40*	(0.12, 0.68)	1357.0	0.24	(−0.03, 0.52)		0.437
Room type: single room	0.46*	(0.10, 0.83)	1575.9	0.40*	(0.05, 0.76)	1726.6	0.818
Manpower: more healthcare professionals	0.13	(−0.07, 0.32)		0.18	(0.00, 0.36)		0.719
Enhanced service: flexible choice of enhanced services	0.25*	(0.05, 0.45)	850.0	0.55*	(0.35, 0.74)	2345.2	0.037*
Copayment (per HK$1000)	−0.29*	(−0.41, − 0.18)		− 0.23*	(− 0.34, − 0.13)		0.438
Opt-out	−2.49*	(−3.13, −1.84)		−3.60*	(−4.57, −2.64)		0.060
	Unmarried older persons (*n* = 216)			Married older persons (*n* = 67)			*P* value^a^
	Coeff.	95%CI	MWTP	Coeff.	95%CI	MWTP	
Type of care homes: NGO care homes	0.36*	(0.15, 0.57)	1532.9	0.56*	(0.18, 0.94)	1540.0	0.358
Distance: half an hour to one hour travelling time	0.18	(−0.02, 0.39)		0.38	(0.00, 0.77)		0.371
Distance: Within half an hour travelling time	0.30*	(0.04, 0.55)	1270.1	0.50*	(0.04, 0.95)	1358.5	0.457
Room type: shared room (2–3 people)	0.35*	(0.14, 0.56)	1476.8	0.47*	(0.09, 0.85)	1285.7	0.577
Room type: single room	0.60*	(0.33, 0.88)	2582.3	0.47	(−0.03, 0.97)		0.647
Manpower: more healthcare professionals	0.16*	(0.01, 0.30)	675.7	0.26	(−0.01, 0.52)		0.526
Enhanced service: flexible choice of enhanced services	0.49*	(0.34, 0.64)	2104.1	0.09	(−0.19, 0.37)		0.012*
Copayment (per HK$1000)	−0.23*	(−0.32, − 0.15)		−0.37*	(− 0.51, − 0.22)		0.131
Opt-out	−2.87*	(−3.46, −2.28)		−2.43*	(−3.27, − 1.59)		0.406
	Caregiver aged below 60 years (*n* = 137)			Caregiver aged 60+ years (*n* = 146)			*P* value^a^
	Coeff.	95%CI	MWTP	Coeff.	95%CI	MWTP	
Type of care homes: NGO care homes	0.42*	(0.14, 0.70)	1514.8	0.39*	(0.15, 0.64)	1509.5	0.905
Distance: half an hour to one hour travelling time	0.54*	(0.26, 0.82)	1965.3	−0.04	(− 0.29, 0.20)		0.002*
Distance: Within half an hour travelling time	0.54*	(0.19, 0.88)	1947.1	0.22	(− 0.07, 0.52)		0.178
Room type: shared room (2–3 people)	0.50*	(0.23, 0.77)	1812.6	0.28*	(0.02, 0.53)	1058.0	0.242
Room type: single room	1.02*	(0.65, 1.40)	3713.6	0.19	(−0.13, 0.52)		0.001*
Manpower: more healthcare professionals	0.35*	(0.17, 0.54)	1280.8	0.03	(−0.14, 0.21)		0.014*
Enhanced service: flexible choice of enhanced services	0.52*	(0.33, 0.72)	1903.1	0.33*	(0.15, 0.51)	1263.2	0.156
Copayment (per HK$1000)	−0.28*	(−0.38, − 0.17)		− 0.26*	(− 0.36, − 0.16)		0.851
Opt-out	−2.60*	(−3.41, −1.80)		−2.85*	(−3.46, −2.24)		0.630

^*^P < 0.05; the reference levels of the attributes are: 1) Type of care homes: private for-profit homes; 2) Distance: Over one hour travelling time; 3) Room type: Shared room (4–6 people); 4) Manpower: more care workers; and 5) Enhanced service: limited choices in enhanced services

^a^The *P* values in this column that were derived from the interaction terms of the regression models indicate the difference of coefficient estimates of the same attribute across different income levels, marital status, or caregiver’s age. The coefficients in the table were calculated by adding the coefficients of the attribute main effects and the coefficients of the interaction terms between the attribute and any of the income level, marital status or caregivers’ age. The 95% confidence intervals were estimated using Delta method. Original model outcomes can be found in supplementary Tables S7, S8 and S9

Second, caregivers of unmarried older persons (*n* = 216) preferred NGO care homes (HK$1532.9), shorter distance (< 0.5-hour travel: HK$1270.1), fewer roommates (single room: HK$2582.3), more healthcare professionals (HK$675.7), and flexible enhanced services (HK$2104.1). On the other hand, caregivers of married older persons (*n* = 67) preferred NGO care homes (HK$1540.0), shorter distance (< 0.5-hour travel: HK$1358.5), and fewer roommates (2–3 people shared room: HK$1285.7). Caregivers of unmarried older persons were found to have significantly greater preference for flexible enhanced services (HK$2104.1 vs insignificant preferences in married group, *P* = 0.012).

Lastly, caregivers aged below 60 years (*n* = 137) preferred NGO care homes (HK$1514.8), shorter distance (< 0.5-hour travel: 1947.1), fewer roommates (single room: HK$3713.6), more healthcare professionals (HK$1280.8), and flexible enhanced services (HK$1903.1), while those aged 60 years or above, preferred NGO care homes (HK$1509.5), 2–3 people shared room (HK$1058.0), and flexible enhanced services (HK$1263.2). Comparing these two groups, caregivers aged below 60 years had greater preferences for shorter travel distance (0.5–1 hour travel: HK$1965.3 vs insignificant preferences, *P* = 0.002), single room (HK$3713.6 vs insignificant preferences, *P* = 0.001), and more healthcare professionals (HK$1280.8 vs insignificant preferences, *P* = 0.014) than those aged 60 years or above.

Table 6 shows the results of the second LCL model. Class 1 (class share: 34.6%) involves those with preference for NGO care homes, shorter distance, and sensitive to the copayment amount. Class 2 (23.2%) involves those with specific preference for NGO care homes. Class 3 (42.2%) involves those with specific preference for flexible choice for enhanced services. Among the individual level characteristics, those with higher income were less likely to be in class 1 than class 3, meaning they tended to have greater preference for flexible enhanced services, which is similar to the finding in MNL model shown in Table 5. Those with lower income tended to prefer NGO care home, shorter distance, and fewer roommates. From the sensitivity analysis (supplementary Tables S4 and S5), caregivers in high income group were less likely to be in class 1 (adjusted difference in probability: -0.06, 95%CI: 0.09–0.03) and class 2 (adjusted difference in probability: -0.01, 95%CI: − 0.02-0.00), while more likely to be in class 3 (adjusted difference in probability: 0.08, 95%CI: 0.05–0.10), which is similar to the LCL model outcome shown in Table 6. In addition, the caregivers aged 60 or above were less likely to be class 3 (adjusted difference in probability: -0.03, 95%CI: 0.06–0.00).

**Table 6:** Preference heterogeneity across income, marital status, and caregiver’s age in latent class model

	Class 1		Class 2		Class 3	
	Coeff.	95%CI	Coeff.	95%CI	Coeff.	95%CI
**Attribute levels**						
Type of care homes: NGO care homes	1.14*	(0.29, 1.99)	2.07*	(1.04, 3.10)	−0.64	(−1.88, 0.61)
Distance: half an hour to one hour travelling time	0.58	(−0.03, 1.18)	0.10	(−0.87, 1.07)	0.20	(−0.26, 0.65)
Distance: Within half an hour travelling time	1.10*	(0.18, 2.02)	0.43	(−1.08, 1.94)	0.30	(−0.42, 1.01)
Room type: shared room (2–3 people)	1.37	(−0.06, 2.81)	0.35	(−0.58, 1.28)	0.14	(−0.47, 0.75)
Room type: single room	1.79	(−0.34, 3.92)	0.27	(−1.03, 1.56)	0.42	(−0.21, 1.05)
Manpower: more healthcare professionals	−0.18	(−0.65, 0.28)	0.61	(−0.38, 1.60)	0.49	(−0.08, 1.07)
Enhanced service: flexible choice of enhanced services	0.14	(−0.96, 1.25)	0.18	(−0.85, 1.20)	1.23*	(0.85, 1.61)
Copayment (per HK$1000)	−1.26*	(−2.46, −0.06)	0.08	(−0.59, 0.76)	0.03	(−0.17, 0.23)
Opt-out	−42.59	-^a^	0.59	(−0.87, 2.05)	−3.11	(−6.95, 0.73)
Class share	0.346		0.232		0.422	
**Membership**						
Communication problem	0.45	(−0.55, 1.45)	0.58	(−1.27, 2.44)	Ref	–
Higher income (HK15,000+)	−0.97*	(−1.89, − 0.05)	−0.39	(− 1.54, 0.75)	Ref	–
Married older person	0.36	(−0.53, 1.25)	0.35	(−0.66, 1.37)	Ref	–
Caregiver aged 60+ years	0.50	(−0.37, 1.37)	0.38	(−0.68, 1.44)	Ref	–
Obs	3396					
Log likelihood	− 662.093					
BIC	1324.187					

^a^The confidence interval was omitted due to large standard error

^*^P < 0.05; the reference levels of the attributes are: 1) Type of care homes: private for-profit homes; 2) Distance: Over one hour travelling time; 3) Room type: Shared room (4–6 people); 4) Manpower: more care workers; and 5) Enhanced service: limited choices in enhanced services

## Discussion

With the launch of RCVS to reduce waiting time and promote choice of LTC, this study examined the preferences of caregivers on the characteristics of the residential care homes, and compared them between the older persons with and without communication problems as defined by MDS-HC. To test hypothesis 1, our results highlighted that the caregivers had greater preference for extra healthcare professionals when the older person did not have communication problems, while they attached higher values to care homes run by NGOs and shorter travelling time to the facility when the older persons had communication problems. This difference might be attributed to the fact that the communication problems of the older persons would increase their perceived needs for more care workers to care for them, as they need more time in interacting with others, as well as more involvement of their family caregivers in daily care service delivery; therefore, NGO care homes and shorter distance of the facility became important to the caregivers. By contrast, availability of healthcare professionals was not as important to older persons with communication problems as to those without the problems.

This finding highlights the importance of quality of care or the reputation of the care homes as well as proximity of the facility to LTC service users with communication problems. The result is comparable to the study in Japan which found that people prefer closer facilities to their current residence if the older adults suffer from dementia, who would likely to have problems in communication functions [33]. As mentioned in the Introduction section, NGO homes had greater average space and staff, as well as a better reputation than private homes. Therefore, this suggests that the private residential care service providers should consider increasing the per-capita manpower and space for the bed places for older persons requiring special attentions to match the standards offered by the NGO care homes, and to promote their facilities among service users by enhancing their liaisons with communities nearby. They should consider establishing collaborations with local community-based elderly centers, or set up community outreach services for older persons and their caregivers, where resources permit. These measures can improve the reputations of their residential care facilities among the residence nearby, and attract more users to consider their facilities when necessary. It is also useful for enhancing the continuity of care for the older persons who usually do not like to relocate to somewhere unfamiliar and without prior social connections [33–35]. Meanwhile, private homes with higher quality of care should be disseminated to the publics to reduce the stereotype, as the LCL model findings on preference heterogeneity suggest that some respondents may also have positive experience in private homes.

Regarding flexibility of choice in care services, previous studies in the UK reported that care home residents with fewer cognitive problems were more likely to benefit from individualized care services than residents with greater cognitive disadvantages, as the latter had limited capacities to exercise their choices [10, 36], and older persons with ADL impairment in Hong Kong were found to have less preference for flexible services [37], while communication problem was not found associated with this preference. However, the ability of those with communication problems to exercise flexible choices is threatened by their limited mental capacity [38], which suggested that there should be personnel and mechanisms in place to assist these residents in optimizing their control and choices over services. Regarding the extra healthcare professionals, the results implied that providing personnel and services to meet social care needs of those with communication problem is as important as those to meet their healthcare needs, while it was found in home care settings that a few healthcare providers tended to leave the psychosocial needs of the older persons with mental impairment to their families [39].

For testing hypothesis 2, preference heterogeneity was examined across income, marital status and caregiver’s age. The financial status and availability of informal support from family members are important factors in decision-making for care homes as reflected by the disparity in WTP according to household income, marital status of older persons and age of caregivers. For household income, the result suggested that less affluent people were more inclined to allocate their financial resources on attributes that can directly improve the living conditions and convenience, including NGO care homes, shorter travel distance and fewer roommates, while more affluent people tended to prefer flexibility of choice in care, which does not seem to benefit the care receipts directly but were found associated with higher satisfaction to services and quality of life [40, 41]. While preferences for various attributes were different between the low income and high income groups, there was no significant difference in the effects of copayment amount on the preference, which took reference from the RCSV scheme co-payment levels that the vast majority of voucher users falls into. This lack of difference across income groups may be attributed to the provision of subsidies in reducing out-of-pocket payments. If there is no such subsidy, the out-of-pocket payment would grow substantially and hence become unaffordable to people with low income, in which case the low income group may become more sensitive to the change in the copayment amount than the high income group.

For marital status, older persons not currently married were less likely to receive sufficient informal support from their family members, and this could potentially lead to greater WTPs in most of the attributes, particularly for the flexible enhanced services, as their caregivers did not have enough time for taking care of them. The pattern is similar among caregivers aged below 60 years, who usually cannot provide enough time for caring as they have not yet retired and they sometimes have their own nuclear family (they are usually adult children of the older persons), despite the fact that they are healthier than caregivers aged over 60 years old. These findings suggested that the lower availability of informal support was associated with greater willingness-to-pay for characteristics indicating higher quality of care, including fewer roommates, extra healthcare professionals, and flexible enhanced services. The importance of informal support is strengthened by evidence in the UK that residents with more supports from family were more likely to benefit from self-directed care services than those without the support [10, 42], implying the necessity of involving available informal support in care planning and even the adjustment of total service fee or co-payment level.

A few limitations in this study should be noted. First, each of the respondents only needed to complete 4 choice tasks, which was lower than 6–8 tasks in many other DCE studies [43–45] and might lead to larger measurement error [46]. However, this number of choice task was determined based on the feedbacks in the pilot study among caregivers, as many of them were also older than 60 years and perceived great burden in providing responses to over 4 choice questions in one interview. Secondly, the sample size in subgroups divided by the DCE blocks, communication problems, income level and availability of informal support is relatively small, which may lead to insufficient power in subgroup analysis. This problem may result in failures and greater random errors in identifying key attributes that influence caregiver’s preference and their differences across subgroups. To reduce the influence of small subgroup sample size, LCL models were used to supplement the MNL models in looking for preferences heterogeneity across subgroups. On the other hand, the non-responders tended to be younger and have less ADL impairment, as younger older persons can respond to the questionnaire by themselves and did not need their caregivers as proxies, who were excluded from the respondents to keep the homogeneity of the study sample as there were only 18 of them. Nevertheless, the influence of this bias is relatively small, as preference heterogeneity was not found across those with and without ADL impairment. Thirdly, this paper focused mainly on caregiver’s preference for care homes, but did not elicit the preference of older persons, as it was difficult for older persons admitted to care homes to complete DCE tasks. However, the disparities in preference between older users of RCSV and caregivers should be aware in care planning for the older persons. Previous studies found that the family proxies were inclined to rate lower importance to activities related to individual hobbies, while they tended to be overprotective and emphasize the safety of the older person in care arrangement [47, 48]. The disparity found between the older persons and caregivers was associated with the relationship between them and the level of family conflicts [20]. Therefore, informational, practical and emotional support from the care professionals should be provided to enable family caregivers to make appropriate decisions that reflect the needs and preference of the older persons [49]. Otherwise, it may lead to a suboptimal choice of care homes for older persons in cases where there is no support for the caregivers and their opinions were the sole criteria in selecting the care homes.

## Conclusions

In summary, caregivers of older persons with communication problems attached greater importance to reputation and proximity of the care homes, while they attached less value to have extra healthcare professionals and a similar value for flexibility of choice in care services. Heterogeneity in preference was substantial among those with different levels of income and informal support. The private care homes should consider establishing collaborations with local community-based elderly centers, or set up community outreach services to older people nearby, to increase their neighborhood reputation and attract more potential users. In addition, the care workers-to-resident ratio should be kept at an appropriate level, while extra health workers are not necessary for those with communication problems. Progressive subsidies should be maintained to narrow the influence of income level on their choices, while availability of informal support should be considered in care planning and even in determining the level of financial subsidies provided.

## Supplementary Information

**Additional file 1: Table S1.** A sample choice set for discrete choice experiment. **Table S2.** Sample characteristics comparison between non-responders and respondents. **Table S3.** Distribution of communication ability across the 15 DCE blocks. **Table S4.** Sensitivity analysis: preference for the care home attributes in latent class model. **Table S5.** Posterior probabilities of class membership and their association with different subgroups. **Table S6.** MNL model outcomes for preferences according to communication problems. **Table S7.** MNL model outcomes for preferences according to household income levels. **Table S8.** MNL model outcomes for preferences according to marital status. **Table S9.** MNL model outcomes for preferences according to caregiver’s age.

## Data Availability

Data used in this study cannot be made publicly available for ethical reasons. Public availability of data would compromise confidentiality and privacy of participants.

## Abbreviations

ADL: Activities of Daily Living
DCE: Discrete choice experiment
DPs: Direct Payments
LTC: Long-term care
MDS-HC: Minimum Data Set – Home Care
NGO: Non-governmental organization
PPP: Public-private partnership
RCSV: Residential care service voucher
WTP: Willingness to pay
