# Supplementary material for: Preference of caregivers on residential care homes for older persons with versus without communication problems: a discrete choice experiment
Source: BMC Geriatr. 2022 May 10;22:411. doi: 10.1186/s12877-022-03073-9 (PMC9087920; doi:10.1186/s12877-022-03073-9)
Supplement: Supplementary file 1 — Additional file 1: Table S1. A sample choice set for discrete choice experiment. Table S2. Sample characteristics comparison between non-responders and respondents. Table S3. Distribution of communication ability across the 15 DCE blocks. Table S4. Sensitivity analysis: preference for the care home attributes in latent class model. Table S5. Posterior probabilities of class membership and their association with different subgroups. Table S6. MNL model outcomes for preferences according to communication problems. Table S7. MNL model outcomes for preferences according to household income levels. Table S8. MNL model outcomes for preferences according to marital status. Table S9. MNL model outcomes for preferences according to caregiver’s age. [file 12877_2022_3073_MOESM1_ESM.docx]

***Supplementary materials.***

**Discrete choice experiment (DCE) sample choice set and supplementary sample characteristics.**

Table S1. A sample choice set for discrete choice experiment

| Hypothetical scenario: assuming that you are considering to move into a subsidized long-term care facility for yourself / the older person you are taking care of in the residential care service voucher scheme (RCSV). You can compare the characteristics of the two care home alternatives (A and B) presented in each of the four choice tasks, and make the decision based on your own preference. The care homes are subsidized by the government, and the co-payment amount is specified as actual out-of-pocket payment for each alternative. If you are not satisfied with both alternatives, you can choose to opt-out by not choosing any care home, meaning you will need to wait 1-3 months for the staff to show you other care homes. There are neither right or wrong answers for your choices. The characteristics that are not mentioned in the tables are identical across the two alternatives. | | | |
| --- | --- | --- | --- |
|  | Residential care home A | Residential care home B | Not choosing any care home |
| Type of care homes | Private-operated (for-profit) care homes | NGO care homes: subvented/ contract/ self-financing care homes | Not choosing any care home: **□** |
| Distance (travelling hour to the facility from home/ office) | Within half an hour | More than an hour |  |
| Room type | Shared room (2-3 people) | Shared room (4 or more people) |  |
| Manpower | Extra healthcare professionals compared with standard requirement | Extra care workers compared with standard requirement |  |
| Enhanced services | Flexibility of choices for enhanced services | Limited choices in enhanced services |  |
| User monthly co-payment amount | HK$2657 | HK$1329 |  |
|  |  |  |  |
| Which residential care home would you like to choose? | Residential care home A: **□** | Residential care home B: **□** |  |

Table S2. Sample characteristics comparison between non-responders and respondents

|  | Non-responders | | Respondents | | Total | | P value |
| --- | --- | --- | --- | --- | --- | --- | --- |
|  | N | % | N | % | N | % |  |
| **Age of older person** | |  |  |  |  |  |  |
| 60-69 | 8 | 7.7 | 8 | 2.8 | 16 | 4.1 | 0.003* |
| 70-79 | 15 | 14.4 | 27 | 9.5 | 42 | 10.9 |  |
| 80-89 | 57 | 54.8 | 128 | 45.2 | 185 | 47.8 |  |
| 90-99 | 24 | 23.1 | 115 | 40.6 | 139 | 35.9 |  |
| 100+ | 0 | 0.0 | 5 | 1.8 | 5 | 1.3 |  |
| (missing) | 2 |  | 0 |  | 2 |  |  |
| **Sex** |  |  |  |  |  |  |  |
| Male | 34 | 32.7 | 82 | 29.0 | 116 | 30.0 | 0.479 |
| Female | 70 | 67.3 | 201 | 71.0 | 271 | 70.0 |  |
| (missing) | 2 |  | 0 |  | 2 |  |  |
| **Activities of daily living** | | |  |  |  |  |  |
| No impairment | 11 | 11.3 | 8 | 2.8 | 19 | 5.0 | 0.001* |
| With impairment | 86 | 88.7 | 275 | 97.2 | 361 | 95.0 |  |
| (missing) | 9 |  | 0 |  | 9 |  |  |
| **Cognition** |  |  |  |  |  |  |  |
| No impairment | 11 | 11.3 | 23 | 8.1 | 34 | 9.0 | 0.339 |
| With impairment | 86 | 88.7 | 260 | 91.9 | 346 | 91.1 |  |
| (missing) | 9 |  | 0 |  | 9 |  |  |
| **Communication problem** | | |  |  |  |  |  |
| No | 31 | 32.0 | 55 | 19.4 | 86 | 22.6 | 0.011* |
| Yes | 66 | 68.0 | 228 | 80.6 | 294 | 77.4 |  |
| (missing) | 9 |  | 0 |  | 9 |  |  |
| **Total** | 106 | 100.0 | 283 | 100.0 | 389 | 100.0 |  |

Table S3. Distribution of communication ability across the 15 DCE blocks

|  | No communication problem | | With communication problem | | Total | | P value |
| --- | --- | --- | --- | --- | --- | --- | --- |
|  | N | % | N | % | N | % |  |
| **DCE block number** | | |  |  |  |  |  |
| 1 | 3 | 5.5 | 16 | 7.0 | 19 | 6.7 | 0.198 |
| 2 | 3 | 5.5 | 16 | 7.0 | 19 | 6.7 |  |
| 3 | 2 | 3.6 | 17 | 7.5 | 19 | 6.7 |  |
| 4 | 7 | 12.7 | 12 | 5.3 | 19 | 6.7 |  |
| 5 | 6 | 10.9 | 14 | 6.1 | 20 | 7.1 |  |
| 6 | 2 | 3.6 | 18 | 7.9 | 20 | 7.1 |  |
| 7 | 5 | 9.1 | 16 | 7.0 | 21 | 7.4 |  |
| 8 | 5 | 9.1 | 18 | 7.9 | 23 | 8.1 |  |
| 9 | 3 | 5.5 | 16 | 7.0 | 19 | 6.7 |  |
| 10 | 7 | 12.7 | 10 | 4.4 | 17 | 6.0 |  |
| 11 | 1 | 1.8 | 17 | 7.5 | 18 | 6.4 |  |
| 12 | 3 | 5.5 | 17 | 7.5 | 20 | 7.1 |  |
| 13 | 2 | 3.6 | 12 | 5.3 | 14 | 5.0 |  |
| 14 | 1 | 1.8 | 15 | 6.6 | 16 | 5.7 |  |
| 15 | 5 | 9.1 | 14 | 6.1 | 19 | 6.7 |  |
| **Total** | 55 | 100.0 | 228 | 100.0 | 283 | 100.0 |  |

Table S4. Sensitivity analysis: preference for the care home attributes in latent class model

|  | Class 1 | | Class 2 | | Class 3 | |
| --- | --- | --- | --- | --- | --- | --- |
|  | Coeff. | 95%CI | Coeff. | 95%CI | Coeff. | 95%CI |
| **Attribute levels** |  |  |  |  |  |  |
| Type of care homes: NGO care homes | 0.84* | (0.58, 1.10) | 0.97 | (-0.16, 2.09) | -1.11* | (-1.98, -0.24) |
| Distance: half an hour to one hour travelling time | 0.40* | (0.19, 0.61) | 0.56 | (-0.70, 1.82) | -0.27 | (-0.92, 0.38) |
| Distance: Within half an hour travelling time | 0.61* | (0.34, 0.89) | 0.79 | (-0.59, 2.16) | -0.28 | (-1.22, 0.67) |
| Room type: shared room (2-3 people) | 0.52* | (0.30, 0.75) | 0.98 | (-0.54, 2.50) | -0.46 | (-1.15, 0.24) |
| Room type: single room | 0.70* | (0.39, 1.01) | 2.16* | (0.56, 3.76) | 0.17 | (-0.77, 1.11) |
| Manpower: more healthcare professionals | -0.02 | (-0.18, 0.14) | 0.37 | (-0.64, 1.37) | 1.69* | (0.41, 2.97) |
| Enhanced service: flexible choice of enhanced services | -0.08 | (-0.26, 0.11) | 0.82 | (-0.24, 1.87) | 2.72* | (1.39, 4.04) |
| Copayment (per HK$1,000) | -0.39* | (-0.48, -0.30) | 0.08 | (-0.34, 0.50) | 0.19 | (-0.07, 0.44) |
| Opt-out | -3.90* | (-4.74, -3.05) | 4.52* | (2.32, 6.72) | -10.86 | (-232.39, 210.67) |
| Class share | 0.624 |  | 0.026 |  | 0.350 |  |
| Obs | 3396 |  |  |  |  |  |
| Log likelihood | -762.608 |  |  |  |  |  |
| BIC | 1525.217 |  |  |  |  |  |

Note: *P<0.05; the reference levels of the attributes are: 1) Type of care homes: private for-profit homes; 2) Distance: Over one hour travelling time; 3) Room type: Shared room (4-6 people); 4) Manpower: more care workers; and 5) Enhanced service: limited choices in enhanced services

Table S5. Posterior probabilities of class membership and their association with different subgroups.

|  | Class 1 probability (median) | Class 2 probability  (median) | Class 3 probability  (median) | Regression coeff. for class 1 | Regression coeff. for class 2 | Regression coeff. for class 3 |
| --- | --- | --- | --- | --- | --- | --- |
| **Communication problem** | |  |  |  |  |  |
| No | 66.6% | 0.008% | 29.9% | Ref | Ref | Ref |
| Yes | 87.6% | 0.013% | 8.9% | 0.07* (0.04, 0.10) | -0.02* (-0.03, -0.01) | -0.05* (-0.08, -0.02) |
| **Monthly household income** | |  |  |  |  |  |
| <HK$ 15,000 | 94.6% | 0.006% | 4.7% | Ref | Ref | Ref |
| HK$ 15,000+ | 72.0% | 0.022% | 23.1% | -0.06* (-0.09, -0.03) | -0.01* (-0.02, 0.00) | 0.08* (0.05, 0.10) |
| **Marital status of the older person** | |  |  |  |  |  |
| Unmarried | 84.8% | 0.020% | 12.3% | Ref | Ref | Ref |
| Married | 94.1% | 0.004% | 4.4% | 0.02 (-0.01, 0.06) | 0.01 (-0.01, 0.02) | -0.03 (-0.06, 0.00) |
| **Caregiver's age** |  |  |  |  |  |  |
| Below 60 years | 79.5% | 0.022% | 17.2% | Ref | Ref | Ref |
| 60+ years | 93.2% | 0.007% | 5.0% | 0.02 (-0.01, 0.05) | 0.01 (0.00, 0.02) | -0.03* (-0.06, 0.00) |

Table S6. MNL model outcomes for preferences according to communication problems

|  | Coeff. | 95%CI |
| --- | --- | --- |
| NGO care home | 0.24 | (-0.15, 0.64) |
| NGO x communication problems | 0.23 | (-0.22, 0.67) |
| 0.5-1 hour travel distance | 0.17 | (-0.25, 0.60) |
| 0.5-1 hour x communication problems | 0.10 | (-0.37, 0.57) |
| <0.5 hour travel distance | 0.15 | (-0.32, 0.62) |
| <0.5 hour x communication problems | 0.25 | (-0.28, 0.79) |
| 2-3 people shared room | 0.63* | (0.20, 1.07) |
| 2-3 people x communication problems | -0.30 | (-0.78, 0.17) |
| Single room | 0.93* | (0.34, 1.51) |
| Single room x communication problems | -0.39 | (-1.03, 0.26) |
| More healthcare professionals | 0.54* | (0.22, 0.85) |
| Healthcare x communication problems | -0.45* | (-0.79, -0.11) |
| Flexible enhanced services | 0.42* | (0.10, 0.74) |
| Flexible x communication problems | -0.01 | (-0.36, 0.34) |
| Copayment (per HK$1000) | -0.30* | (-0.47, -0.14) |
| Copayment x communication problems | 0.04 | (-0.15, 0.22) |
| Opt-out | -2.27* | (-3.30, -1.25) |
| Opt-out x communication problems | -0.54 | (-1.70, 0.63) |

Table S7. MNL model outcomes for preferences according to household income levels

|  | Coeff. | 95%CI |
| --- | --- | --- |
| NGO care home | 0.41* | (0.14, 0.69) |
| NGO x income level | -0.08 | (-0.47, 0.31) |
| 0.5-1 hour travel distance | 0.20 | (-0.08, 0.48) |
| 0.5-1 hour x income level | -0.07 | (-0.46, 0.32) |
| <0.5 hour travel distance | 0.50* | (0.17, 0.82) |
| <0.5 hour x income level | -0.39 | (-0.86, 0.08) |
| 2-3 people shared room | 0.40* | (0.12, 0.68) |
| 2-3 people x income level | -0.16 | (-0.55, 0.24) |
| Single room | 0.46* | (0.10, 0.83) |
| Single room x income level | -0.06 | (-0.57, 0.45) |
| More healthcare professionals | 0.13 | (-0.07, 0.32) |
| Healthcare x income level | 0.05 | (-0.22, 0.32) |
| Flexible enhanced services | 0.25* | (0.05, 0.45) |
| Flexible x income level | 0.30* | (0.02, 0.58) |
| Copayment (per HK$1000) | -0.29* | (-0.41, -0.18) |
| Copayment x income level | 0.06 | (-0.09, 0.21) |
| Opt-out | -2.49* | (-3.13, -1.84) |
| Opt-out x income level | -1.11 | (-2.28, 0.05) |

Table S8. MNL model outcomes for preferences according to marital status

|  | Coeff. | 95%CI |
| --- | --- | --- |
| NGO care home | 0.36* | (0.15, 0.57) |
| NGO x marital status | 0.20 | (-0.23, 0.64) |
| 0.5-1 hour travel distance | 0.18 | (-0.02, 0.39) |
| 0.5-1 hour x marital status | 0.20 | (-0.24, 0.64) |
| <0.5 hour travel distance | 0.30* | (0.04, 0.55) |
| <0.5 hour x marital status | 0.20 | (-0.33, 0.72) |
| 2-3 people shared room | 0.35* | (0.14, 0.56) |
| 2-3 people x marital status | 0.12 | (-0.31, 0.56) |
| Single room | 0.60* | (0.33, 0.88) |
| Single room x marital status | -0.13 | (-0.71, 0.44) |
| More healthcare professionals | 0.16* | (0.01, 0.30) |
| Healthcare x marital status | 0.10 | (-0.20, 0.40) |
| Flexible enhanced services | 0.49* | (0.34, 0.64) |
| Flexible x marital status | -0.40* | (-0.72, -0.09) |
| Copayment (per HK$1000) | -0.23* | (-0.32, -0.15) |
| Copayment x marital status | -0.13 | (-0.30, 0.04) |
| Opt-out | -2.87* | (-3.46, -2.28) |
| Opt-out x marital status | 0.44 | (-0.59, 1.47) |

Table S9. MNL model outcomes for preferences according to caregiver’s age

|  | Coeff. | 95%CI |
| --- | --- | --- |
| NGO care home | 0.42* | (0.14, 0.70) |
| NGO x caregivers’ age | -0.02 | (-0.39, 0.35) |
| 0.5-1 hour travel distance | 0.54* | (0.26, 0.82) |
| 0.5-1 hour x caregivers’ age | -0.58* | (-0.96, -0.21) |
| <0.5 hour travel distance | 0.54* | (0.19, 0.88) |
| <0.5 hour x caregivers’ age | -0.31 | (-0.77, 0.14) |
| 2-3 people shared room | 0.50* | (0.23, 0.77) |
| 2-3 people x caregivers’ age | -0.22 | (-0.60, 0.15) |
| Single room | 1.02* | (0.65, 1.40) |
| Single room x caregivers’ age | -0.83* | (-1.32, -0.33) |
| More healthcare professionals | 0.35* | (0.17, 0.54) |
| Healthcare x caregivers’ age | -0.32* | (-0.58, -0.07) |
| Flexible enhanced services | 0.52* | (0.33, 0.72) |
| Flexible x caregivers’ age | -0.19 | (-0.46, 0.07) |
| Copayment (per HK$1000) | -0.28* | (-0.38, -0.17) |
| Copayment x caregivers’ age | 0.01 | (-0.13, 0.16) |
| Opt-out | -2.60* | (-3.41, -1.80) |
| Opt-out x caregivers’ age | -0.25 | (-1.26, 0.76) |
